# Supplementary material for: Rapid Nanopore Sequencing of Positive Blood Cultures Using Automated Benzyl-Alcohol Extraction Improves Time-Critical Sepsis Management
Source: Antibiotics (Basel). 2025 Oct 9;14(10):1001. doi: 10.3390/antibiotics14101001 (PMC12561388; doi:10.3390/antibiotics14101001)
Supplement: Supplementary file 1 [file antibiotics-14-01001-s001.zip › Supplementary Figure S1.pdf]

Supplementary Figure S1. Distribution of per-read quality scores.

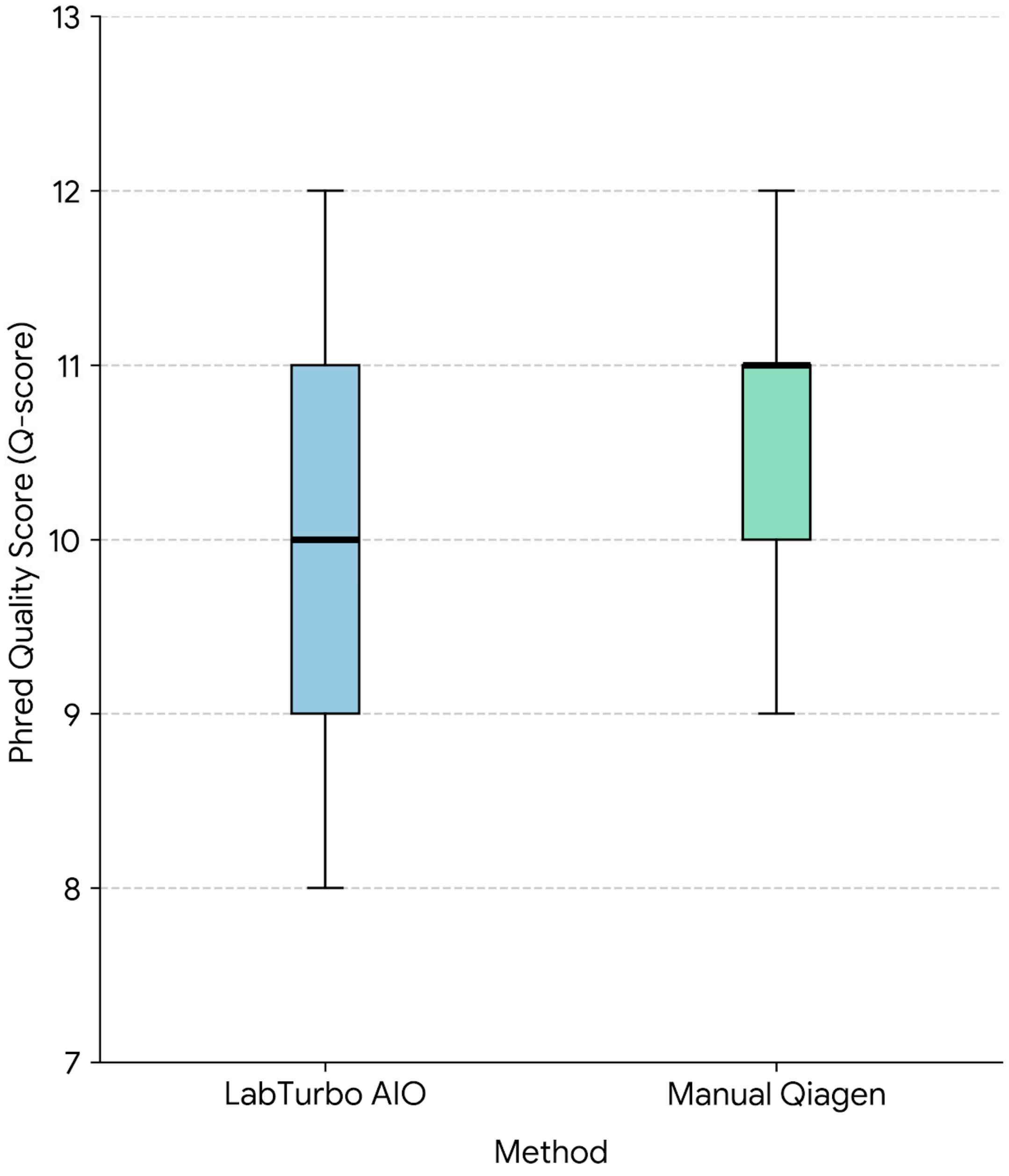

Figure: Per-sample read quality (Q score) distributions for LabTurbo AIO vs. Qiagen. Boxes show the interquartile range with medians; means are indicated; paired sample values are overlaid with connecting segments.
